# Genomic relationships based on X chromosome markers and accuracy of genomic predictions with and without X chromosome markers

**DOI:** 10.1186/1297-9686-46-47

**Published:** 2014-07-30

**Authors:** Guosheng Su, Bernt Guldbrandtsen, Gert P Aamand, Ismo Strandén, Mogens S Lund

**Affiliations:** 1 Center for Quantitative Genetics and Genomics, Department of Molecular Biology and Genetics, Aarhus University, Tjele DK-8830, Denmark; 2 Nordic Cattle Genetic Evaluation, Aarhus N DK-8200, Denmark; 3 Biotechnology and Food Research, MTT Agrifood Research, Jokioinen 31600, Finland

## Abstract

**Background:**

Although the X chromosome is the second largest bovine chromosome, markers on the X chromosome are not used for genomic prediction in some countries and populations. In this study, we presented a method for computing genomic relationships using X chromosome markers, investigated the accuracy of imputation from a low density (7K) to the 54K SNP (single nucleotide polymorphism) panel, and compared the accuracy of genomic prediction with and without using X chromosome markers.

**Methods:**

The impact of considering X chromosome markers on prediction accuracy was assessed using data from Nordic Holstein bulls and different sets of SNPs: (a) the 54K SNPs for reference and test animals, (b) SNPs imputed from the 7K to the 54K SNP panel for test animals, (c) SNPs imputed from the 7K to the 54K panel for half of the reference animals, and (d) the 7K SNP panel for all animals. Beagle and Findhap were used for imputation. GBLUP (genomic best linear unbiased prediction) models with or without X chromosome markers and with or without a residual polygenic effect were used to predict genomic breeding values for 15 traits.

**Results:**

Averaged over the two imputation datasets, correlation coefficients between imputed and true genotypes for autosomal markers, pseudo-autosomal markers, and X-specific markers were 0.971, 0.831 and 0.935 when using Findhap, and 0.983, 0.856 and 0.937 when using Beagle. Estimated reliabilities of genomic predictions based on the imputed datasets using Findhap or Beagle were very close to those using the real 54K data. Genomic prediction using all markers gave slightly higher reliabilities than predictions without X chromosome markers. Based on our data which included only bulls, using a **G** matrix that accounted for sex-linked relationships did not improve prediction, compared with a **G** matrix that did not account for sex-linked relationships. A model that included a polygenic effect did not recover the loss of prediction accuracy from exclusion of X chromosome markers.

**Conclusions:**

The results from this study suggest that markers on the X chromosome contribute to accuracy of genomic predictions and should be used for routine genomic evaluation.

## Background

According to the UMD 3.1 assembly, chromosome X is the second largest chromosome in the bovine genome [1]. A total of 1128 annotated genes have been reported on the X chromosome in the ENSEMBL version 72 [2]. However, markers on the X chromosome are not used for genomic prediction in some countries and populations. Previously, Nordic genomic evaluations used X chromosome markers for genomic predictions in Nordic Red and Jersey populations but not in the Holstein population because markers on the X chromosome were not included in the EuroGenomics project [3].

In mammals, inheritance of chromosome X differs from inheritance of autosomes. In cattle, a sire passes its X chromosome to all its daughters but never to its sons. Consequently, a male inherits a copy of the X chromosome from its mother only, while a female inherits one copy of the X chromosome from its father and one copy from its mother. Therefore, the relationships caused by the X chromosome are different for males and females. Furthermore, a small region of the X chromosome, called the pseudo-autosomal region (PAR) is homologous to the Y chromosome and is inherited in an autosome-like fashion. This increases the complexity of the genetic relationships between individuals based on the X chromosome. Moreover, in genomic prediction of dairy cattle, deregressed proofs (DRP), daughter yield deviations (DYD) and estimated breeding values (EBV) are usually used as response variables. These variables are predicted using a model in which a pedigree-based relationship matrix is constructed based on inheritance of autosomes. In addition, the density of markers on the X chromosome is markedly lower than that on the autosomes in the current SNP (single nucleotide polymorphism) chips [4,5]. These characteristics may reduce the impact of X chromosome markers on accuracy of genomic prediction, and could be the reason why they are not used for genomic prediction in some countries and populations.

Based on the characteristics of the X chromosome, it can be hypothesized that X chromosome markers can contribute to the accuracy of genomic predictions, but will generally have a smaller impact than autosomal markers. Moreover, genomic prediction using a genomic relationship matrix that takes sex-linked inheritance for X-specific markers into account will probably perform better than using a genomic relationship matrix that does not distinguish between autosomal and X-specific markers. In addition, because marker density is lower on the X chromosome, imputation of X chromosome markers may be less accurate than that of autosomal markers. When genomic predictions are performed using data from SNP chips with different densities, genotypes of SNPs absent from low-density chips are usually inferred (imputed) from the higher density chips. Therefore, it is necessary to investigate the accuracy of imputation of markers on the X chromosome in order to perform genomic prediction using these markers. However, so far there are very few reports on the imputation accuracy of X chromosome markers [6] and on their contribution to accuracy of genomic predictions [7].

The objectives of this study were (i) to investigate the accuracy of imputing missing genotypes on the X chromosome, (ii) to demonstrate a method to calculate a genomic relationship matrix which correctly accounts for genetic relationships with regard to markers on the X chromosome, and (iii) to compare the accuracy of genomic predictions with and without X chromosome information using different models and different scenarios. Data from Nordic Holstein cattle were used to address these objectives.

## Methods

### Data

The data used in this analysis consisted of 5643 progeny-tested Nordic Holstein bulls born from 1974 to 2010. The data did not include cows since the number of Nordic Holstein cows available as reference animals was insufficient for the present analysis. Animals were genotyped with the Illumina Bovine SNP50 BeadChip [4]. In order to investigate the accuracy of imputation for markers on the X chromosome, low-density (LD) marker data were created from the SNP50 BeadChip marker data by masking markers that are absent from the Illumina BovineLD BeadChip [5]. The Bovine SNP50 BeadChip (about 54K) and the BovineLD BeadChip (about 7K) marker data were edited by removing markers with a minor allele frequency (MAF) lower than 0.01, an average GenCall score lower than 0.60, or an unknown location in UMD 3.1 [1]. After editing, 44 141 markers remained in the 54K data, and 6699 markers in the LD data. The numbers of markers available on the autosomes and on the X chromosome are in Table 1.

**Table 1:** Number of SNPs used after editing (MAF > 0.01, average GC score > 0.60)

**Marker data**	**Autosomes**	**X chromosome**	
		**PAR**^**a**^	**X-specific**
54K	43 314	133	694
LD (7K)	6458	25	188

^a^PAR: pseudo-autosomal region on the X chromosome.

The bulls were divided into a reference population and a test population according to birth date, i.e., 3995 bulls born before January 1 2005 constituted the reference population and the remaining 1648 bulls constituted the test population. Four sets of data were used to validate accuracies of genotype imputation and genomic prediction: (1) 54K_real: all animals had marker data from the 54K chip; (2) IMP_test: for the test animals, the 54K marker data were imputed from LD marker data; (3) IMP_0.5ref: for half (randomly chosen) of the reference animals, the 54K marker data were imputed from LD marker data, and (4) LD_real: all animals had LD marker data without imputation to the 54K marker data.

The phenotypic data for genomic prediction were DRP that were derived from the Nordic genetic evaluations of January 2013. Fifteen traits included in the Nordic Total Merit index (http://www.nordicebv.info) were analyzed. DRP with reliabilities lower than 10% for animals in the reference data and lower than 20% for animals in the test data were deleted. The number of animals with phenotypic information differed between traits because the number of bulls with published EBV differed between traits. The number of animals available for genomic prediction and the heritability (provided by Nordic Cattle Genetic Evaluation) for each trait are in Table 2.

**Table 2:** Number of animals in the reference data and the test data, and heritability of the traits studied

**Traits**	**Reference data**	**Test data**	**Heritability**
Milk	3943	1159	0.39
Fat	3943	1159	0.39
Protein	3943	1159	0.39
Growth	3451	1351	0.30
Fertility	3975	1158	0.04
Birth index	3988	1642	0.06
Calving index	3986	1239	0.03
Udder health	3987	1204	0.04
Other diseases	3961	1050	0.02
Body conformation	3823	1156	0.30
Feet and legs	3864	1150	0.10
Udder conformation	3866	1156	0.25
Milking ability	3832	1155	0.26
Temperament	3856	1142	0.13
Longevity	3943	817	0.10
**Average**	**3891**	**1180**	**0.19**

### Imputation methods

For datasets IMP_test and IMP_0.5ref, the LD marker data were imputed to the 54K data using two programs: Beagle version 3.3.1 [8] and Findhap version 2 [9]. Beagle uses population information and a hidden Markov model to impute missing genotypes. Findhap is a fast program that imputes missing genotypes using both family and population information and takes the inheritance pattern of the X chromosome into account. Therefore, when using Findhap, markers on the PAR of the X chromosome were treated as autosomal markers, while the rest were treated as X-specific markers. The PAR was approximately identified based on the region of the X chromosome where markers had a substantial proportion of heterozygous genotypes (H%) in the genotyped bulls. The starting position of the region was determined with the criteria that the H% at a SNP was higher than 5%, and at least five of the following 10 SNPs with a MAF larger than 0.05 had a H% higher than 5%. The PAR stopped at the end of the X chromosome. For datasets 54K_real and LD_real, sporadic missing genotypes (4%) were imputed using Beagle.

Genotypes for the imputed markers (in datasets IMP_test and IMP_0.5ref) were compared to their corresponding real genotypes in 54K_real. Accuracy of imputation was measured by the ratio of the number of falsely imputed alleles to total number of imputed alleles, which will be referred to as allele error rate and the ratio of the number of falsely imputed genotypes to the total number of imputed genotypes, which will be referred to as genotype error rate, as well as the correlation between imputed and true genotypes.

### Genomic relationship matrix (G matrix) using marker data including X-specific markers

As presented by VanRaden [10] and Hayes et al. [11], a genomic relationship matrix (**G**) can be calculated as:

G=MM'/∑2pj1-pj,

where elements in column *j* (*m*_*ij*_) of **M** are 0 - 2*p*_*j*_, 1 - 2*p*_*j*_ and 2 - 2*p*_*j*_ for SNP genotypes A_1_A_1_, A_1_A_2_ and A_2_A_2_, respectively, *p*_*j*_ is the frequency of allele A_2_ at SNP *j*. The **G** matrix is calculated based on identity by state (IBS), with centering and scaling. Consequently, elements of the **G** matrix are approximations of realized proportions of the genome that are identical by descent (IBD) between pairs of individuals [11], which makes the **G** matrix analogous to the conventional numerator relationship matrix [10].

The **G** matrix describes the realized genetic relationships between pairs of individuals at the autosomal markers. However, genetic relationships between individuals at markers on the sex chromosomes and the autosomes are different. For example, for markers on the X-specific region of the X chromosome, the genetic relationship is 0 between father and son, 12 between mother and son and between father and daughter, 0.50 between mother and daughter and between full brothers, 0.75 between full sisters, and 12×0.50 between full brother and sister. For autosomal loci, these relationships all have an expectation of 0.50. Therefore, sex-linked inheritance should be considered when building a genomic relationship matrix based on marker data that include X chromosome markers.

When X-specific markers are treated as autosomal markers, the resulting genomic relationship matrix reflects sex-linked relationships, but on an incorrect scale because males have one X chromosome while females have two. For example, the relationship between sire and son is 0, but the diagonal element for a male is 2, instead of 1. Consequently, the covariance structures for males, for females, and between males and females differ from each other.

Let A_1_O and A_2_O denote genotypes of an X-specific marker in males (O means null, since males have only one X chromosome), and A_1_A_1_, A_1_A_2_ and A_2_A_2_ denote genotypes in females. Assuming that A_i_O in males has the same effect on the performance of a trait as A_i_A_i_ in females, genotypes of an X-specific marker can be coded in the same way as autosomal markers. Thus, genotypes A_1_O and A_2_O of males are coded as 0 and 2, and genotypes A_1_A_1_, A_1_A_2_ and A_2_A_2_ of females are coded as 0, 1 and 2. In addition, define γ as the effect of A_2_ (i.e., allele effect on performance of a trait is expressed as the deviation from the effect of A1, thus the effect of A1 is zero), *p* as the frequency of A_2,_ and *q* = 1-*p*. The expectation of the genetic value (*μ*) accounted for by an X-specific marker for a male is:

$$\mu =\left(q\times 0+p\times 2\right)\gamma =2p\gamma .$$

Let *x* be the genotype code as defined above and assume that the allele effect is independent of allele frequency and is additive (i.e., absence of non-additive genetic effect), then the variance of genetic value (*σ*^*2*^) at an X-specific locus in the population of males is:

$$\begin{array}{l} \sigma^{2}=\text{Var}\left(\left(x-2p\right)\gamma \right)\;\\ =\mathrm{Var}\left(x-2p\right)\sigma_{\gamma }^{2}\;\\ =\left[q{\left(0-2p\right)}^{2}+p{\left(2-2p\right)}^{2}\right]\sigma_{\gamma }^{2,}\\ =4\mathrm{pq}\sigma_{\gamma }^{2}\; \end{array}$$

where $\sigma_{\gamma }^{2}$ is the variance of the random additive allele effect *γ*.

For females, the expectation and variance are the same as those for autosomal markers, i.e.

μ=2pγ,

and

$$\sigma^{2}=2\mathrm{pq}\sigma_{\gamma }^{2}.$$

Let *m*_*ij*_ be the element of matrix **M** for individual *i* and marker *j*, as defined previously. The relationship coefficient between male *k* and male *l* caused by the X-specific marker *j* can then be calculated as:

$$r_{\mathrm{kl}}=m_{\mathrm{kj}}m_{\mathrm{lj}}/4p_{j}q_{j}.$$

The relationship coefficient between female *k* and female *l* has the same form as for autosomal markers, i.e.

$$r_{\mathrm{kl}}=m_{\mathrm{kj}}m_{\mathrm{lj}}/2p_{j}q_{j}.$$

The relationship coefficient between male *k* and female *l* is:

$$r_{\mathrm{kl}}=m_{\mathrm{kj}}m_{\mathrm{lj}}/\sqrt{4p_{j}q_{j}2p_{j}q_{j}}.$$

Alternatively, it can be assumed that genotype A_i_O in males has half the effect of genotype A_i_A_i_ in females. Then, the genotypes can be coded as the number of copies of A_2_, i.e., 0 and 1 for genotypes A_1_O and A_2_O of males, 0, 1 and 2 for genotypes A_1_A_1_, A_1_A_2_ and A_2_A_2_ of females, respectively. For females, the expectation and variance accounted for by an X-specific marker are the same as the above. The expectation of the genetic value for a male is:

$$\mu =\left(q\times 0+p\times 1\right)\gamma =p\gamma ,$$

and the variance of the genetic value for males is:

$$\begin{array}{ll} \sigma^{2} & =\left[q{\left(0-p\right)}^{2}+p{\left(1-p\right)}^{2}\right]\sigma_{\gamma }^{2}\\ & =\mathrm{pq}\sigma_{\gamma }^{2}\;. \end{array}$$

Let m^*^_ij_ be the element for individual *i* and marker *j* in the corresponding **M** matrix. Define m^*^_ij_ = 0-*p* for genotype A_1_O and m^*^_ij_ = 1-*p*_*j*_ for genotype A_2_O of males, and m^*^_ij_ = 0-2*p*_*j*_, 1-2*p*_*j*_ or 2-2*p*_*j*_ for genotypes A_1_A_1_, A_1_A_2_, or A_2_A_2_ of females. Then, m^*^_ij_ = *m*_*ij*_/2 for males, and m^*^_ij_ = *m*_*ij*_ for females.

Then, the relationship coefficient between male *k* and male *l* caused by the X-specific marker *j* is:

$$r_{\mathrm{kl}}={m^{*}}_{\mathrm{kj}}{m^{*}}_{\mathrm{lj}}/p_{j}q_{j}=m_{\mathrm{kj}}m_{\mathrm{lj}}/4p_{j}q_{j},$$

the relationship coefficient between female *k* and female *l* is:

$$r_{\mathrm{kl}}={m^{*}}_{\mathrm{kj}}{m^{*}}_{\mathrm{lj}}/2p_{j}q_{j}=m_{\mathrm{kj}}m_{\mathrm{lj}}/2p_{j}q_{j},$$

and the relationship coefficient between male *k* and female *l* is:

$$r_{\mathrm{kl}}={m^{*}}_{\mathrm{kj}}{m^{*}}_{\mathrm{lj}}/\sqrt{p_{j}q_{j}2p_{j}q_{j}}=m_{\mathrm{kj}}m_{\mathrm{lj}}/\sqrt{4p_{j}q_{j}2p_{j}q_{j}}.$$

This demonstrates that the two alternate assumptions for the effect of the male genotype of X-specific markers lead to the same relationship coefficient. Thus, the **G** matrix based on both autosomal and X chromosome markers can be calculated as for autosomal markers, but element *m*_*ij*_ of the **M** matrix must be divided by $\sqrt{2}\;$ if marker *j* is a X-specific marker and individual *i* is a male, i.e.

$$\text{new}\;m_{j}=\left\{\begin{array}{l} m_{j}\circ \delta \;\mathrm{if}\;j\;\mathrm{is}\;\mathrm{an}\;X‒\text{specific}\;\text{marker}\\ m_{j}\;\text{else}\; \end{array}\right)\;,$$

where ° is the Hadamard product operation, element *i* in vector **δ** is 1 if individual *i* is a female, and 12 if individual *i* is a male. To construct the **M** matrix, when the codes for A_1_A_1_, A_1_A_2_ and A_2_A_2_ are 0, 1 and 2, the X-specific genotypes of A_1_O and A_2_O are coded as 0 and 2.

### Genomic prediction models

Genomic predictions based on marker data with and without markers on the X chromosome were carried out using the following GBLUP models implemented in the DMU package [12]:

(1) G(A): GBLUP with the **G** matrix built using autosomal markers (**G**_**a**_) only:

$$y=\mu +Zg_{a}+e;$$

(2) G(A + X): GBLUP with the **G** matrix built using all markers and treating X-specific markers as autosomal markers (**G**_**0**_):

$$y=\mu +Zg_{0}+e;$$

(3) G_c_(A + X): GBLUP with the **G** matrix built using all markers and accounting for the sex-linked inheritance of X-specific markers (**G**_**c**_),

$$y=\mu +Zg_{c}+e;$$

(4) G(A) + G(X): GBLUP using both the autosomal **G** matrix and the X chromosome **G** matrix (**G**_**x**_):

$$y=\mu +Zg_{a}+Zg_{x}+e;$$

(5) G(A) + Pol: model G(A) plus a residual polygenic effect:

$$y=\mu +Zg_{a}+Z_{u}u+e;$$

(6) G_c_(A + X) + Pol: model G_c_(A + X) plus a residual polygenic effect:

$$y=\mu +Zg_{c}+Z_{u}u+e.$$

In the above models, **y** is the vector of DRP, μ is the intercept, **g**_a_ is the vector of genomic breeding values accounted for by autosomes, **g**_x_ is the vector of genomic breeding values accounted for by the X chromosome, **g**_0_ is the vector of total genomic breeding values associated with the **G** matrix that treats X-specific markers as autosomal markers, **g**_**c**_ is the vector of total genomic breeding values associated with the **G** matrix that accounts for X-specific markers as sex-linked markers, **Z** is the incidence matrix relating genomic breeding values to **y**, **u** is the vector of residual polygenic effects, **Z**_u_ is the incidence matrix that associates **u** with **y**, and **e** is the vector of random residuals. Random effects are assumed distributed as follows:

$$\begin{array}{l} g_{a}\sim N\left(0,G_{a}\sigma_{g_{a}}^{2}\right),\;g_{0}\sim N\left(0,G_{0}\sigma_{g_{0}}^{2}\right),\;g_{c}\sim N\left(0,G_{c}\sigma_{g_{c}}^{2}\right),\;\\ g_{x}\sim N\left(0,G_{x}\sigma_{g_{x}}^{2}\right),\;u\sim N\left(0,A\sigma_{u}^{2}\right),\; \end{array}$$

$$\mathrm{and}\;e\sim N\left(0,R\sigma_{e}^{2}\right),$$

where **A** is the pedigree-based relationship matrix, and **R** is a diagonal matrix used to account for heterogeneous residual variances due to different reliabilities of DRP ($r_{\mathrm{DRP}}^{2}$). The diagonal element *i* of matrix **R** was computed as $R_{\mathrm{ii}}=\frac{1-r_{\mathrm{DRP}}^{2}}{r_{\mathrm{DRP}}^{2}}$. Reliability of DRP was calculated as $r_{\text{DRP}}^{2}=\frac{\text{EDC}}{\text{EDC}+\lambda }$ where EDC is the equivalent daughter contribution and $\lambda =\frac{4-\text{heritability}}{\text{heritability}}$[13]. All variances ($\sigma_{g_{a}}^{2},\;\sigma_{g_{x}}^{2},\;\sigma_{g_{0}}^{2},\;\sigma_{g_{c}}^{2},\;\sigma_{u}^{2},\;$ and $\sigma_{e}^{2}$) were estimated from the DRP data used in the analyses, using the corresponding models. The allele frequencies used to construct the **G** matrix were calculated from the current marker data of the genotyped animals.

In addition to the above analyses, genomic predictions were also performed using four reduced 54K marker datasets. These datasets were: (1) Non-2: marker data excluding the markers on chromosome 2 that has a length similar to that of the X chromosome; (2) Non-10: marker data excluding the markers on chromosome 10 which is similar to the X chromosome in terms of number of annotated genes; (3) Non-26: marker data excluding the markers on chromosome 26 which is similar to X chromosome in terms of number of markers; (4) Non-ran: marker data excluding a random sample of 827 markers (equivalent to the number of markers available on the X chromosome). Genomic predictions based on these datasets were carried out using the GBLUP model **y** = μ + **Zg**_r_ + **e**,  where **g**_r_ is the vector of genomic breeding values accounted for by the reduced marker data. The **G** matrix used for the analyses considered sex-linked inheritance for X-specific markers.

Genomic predictions using different marker datasets and different models were validated by comparing genomic estimated breeding values (GEBV) and DRP for animals in the test data. GEBV were calculated as the sum of the genomic effect and the residual polygenic effect for models G(A) + Pol and G_c_(A + X) + Pol, and as the sum of the autosomal effect and the X chromosome effect for model G(A) + G(X). Reliabilities of genomic predictions were estimated as the squared correlation between genomic predictions and DRP, and then divided by the average reliability of DRP, based on [14]:

$$\begin{array}{ll} r_{\text{GEBV}}^{2} & =\frac{{\text{Cov}}^{2}\left(\text{GEBV},\text{DRP}\right)}{\sigma_{\text{GEBV}}^{2}\sigma_{\text{DRP}}^{2}r_{\text{DRP}}^{2}}\;\\ & =\frac{{\text{Cov}}^{2}\left(\text{GEBV},\text{TBV}+\text{residual}\right)}{\sigma_{\text{GEBV}}^{2}\sigma_{\text{TBV}}^{2}},\\ & =\frac{{\text{Cov}}^{2}\left(\text{GEBV},\text{TBV}\right)}{\sigma_{\text{GEBV}}^{2}\sigma_{\text{TBV}}^{2}} \end{array}$$

where TBV is true breeding value. Bias of genomic predictions was assessed by regression of DRP on GEBV [15]. A necessary condition for unbiased prediction is that the regression coefficient does not deviate significantly from 1.

The log-likelihood ratio statistic (-2lnLR) was used to test the difference in goodness of fit between model G(A) + G(X) and model G(A), and between model G_c_(A + X) + Pol and model G_c_(A + X). Taking G(A) + G(X) and G_c_(A + X) + Pol as alternative model while G(A) and G_c_(A + X) as null model, the log-likelihood ratio statistic was calculated as -2lnLR = -2ln(likelihood of null model/likelihood of alternative model). The P value of -2lnLR was calculated assuming that -2lnLR is asymptotically $\chi_{\mathrm{df}=1}^{2}$ distributed [16], and calculated assuming that the asymptotic distribution of -2lnLR is a 50:50 mixture of $\chi_{\mathrm{df}=0}^{2}$ and $\chi_{\mathrm{df}=1}^{2},\;$ so that P(-$\chi_{\mathrm{mixture}}^{2}$) = 0.5P ($\chi_{\mathrm{df}=1}^{2}$) [17]. Hotelling-Williams’ t-test [18,19] was implemented to test the equality of two dependent correlations (Cor(GEBV, DRP)) from two models for the same trait. The log-likelihood ratio test and Hotelling-Williams’ t-test were implemented in the analysis using the 54K_real marker data .

## Results

The accuracy of imputation from the 7K to the 54K SNP panel was high (Table 3). Using Beagle, the allele error rate for autosomal markers averaged over the two datasets (IMP_test and IMP_0.5ref) was 1.1%. Compared with autosomal markers, the allele error rates for X-specific markers and PAR markers were increased by 2.1 and 7.7%, respectively. The accuracy of imputation with Findhap was slightly lower than that with Beagle, with an increase of the allele error rate of about 0.7% for autosomes, 0.3% for X-specific markers, and 1.5% for PAR markers, averaged over the two datasets. Correlation coefficients between imputed and true genotypes for autosomal markers, pseudo-autosomal markers, and X-specific markers were 0.983, 0.856 and 0.937 with Beagle, and 0.971, 0.831 and 0.935 with Finhap.

**Table 3:** **Allele error rate (ER**_**A**_**, %), genotype error rate (ER**_**G**_**, %) and correlation (COR) between imputed and true genotypes for different sets of markers**^**a **^**in two datasets**^**b**^

**Dataset**	**Method**	**ALL**			**AUTO**			**PAR**			**X**		
		**ER**_**A**_	**ER**_**G**_	**COR**	**ER**_**A**_	**ER**_**G**_	**COR**	**ER**_**A**_	**ER**_**G**_	**COR**	**ER**_**A**_	**ER**_**G**_	**COR**
IMP_test	Findhap	1.7	3.3	0.972	1.7	3.3	0.974	10.4	19.1	0.829	3.3	4.1	0.940
	Beagle	1.1	2.2	0.982	1.1	2.1	0.983	8.8	15.9	0.858	3.0	3.0	0.941
IMP_0.5ref	Findhap	2.0	3.9	0.967	2.0	3.8	0.968	10.3	18.7	0.833	3.8	4.4	0.930
	Beagle	1.2	2.4	0.981	1.2	2.3	0.982	8.9	16.4	0.854	3.5	3.9	0.933

^a^ALL: all markers; AUTO: markers on the autosomes; PAR: markers on the pseudo-autosomal region; X: X-specific markers on the X chromosome; ^b^IMP_test: for the test animals in genomic prediction, the 54K marker data were imputed from LD marker data; IMP_0.5ref: for half (randomly chosen) of the reference animals, the 54K marker data were imputed from LD marker data.

Genotype error rate was nearly twice as large as the allele error rate for markers on autosomes and PAR, but almost the same for X-specific markers (Table 3). This was because animals in the present data were all bulls, thus genotype error was in principle equivalent to the allele error for X-specific markers. The reason for a slightly higher genotype error rate than allele error rate for X-specific markers was that some genotypes were heterozygous in the real 54K data (due to typing error) and in the imputed data (due to imputation error).

Although animals with LD genotypes in the IMP_test dataset had more ancestors with 54K genotypes, while animals with LD genotypes in the IMP_0.5ref dataset had more progeny with 54K genotypes, these two datasets had similar accuracies of imputation (Table 3). Allele error rates were equal to 1.9% with Findhap and 1.2% with Beagle, averaged over the two imputation datasets and calculated from the data pooled over the autosomes and the X chromosome markers.

As shown in Table 4, for the four datasets, genomic predictions using all markers gave a slightly higher reliability than predictions without markers on the X chromosome. Averaged over the 15 traits, the gain in reliability from using the X chromosome markers was 0.4 to 0.5% points when using models without a residual polygenic effect, and 0.3 to 0.4% points when using models with a residual polygenic effect. Models G(A + X) and G_c_(A + X) resulted in the same reliability of genomic predictions, which indicates that a **G** matrix that took sex-linked inheritance for X-specific markers into account did not improve genomic prediction more than a **G** matrix that dealt with X-specific markers as autosomal markers, possibly because animals in the present data were all bulls. In addition, model G(A) + G(X) did not improve predictions compared to models G(A + X) and G_c_(A + X), which suggests that it is reasonable to assume that the effects of the markers on the X chromosome and the autosomes have the same distribution.

**Table 4:** **Reliability (%) of genomic predictions based on four datasets**^**a **^**with or without X chromosome markers, using different models**^**b **^**and averaged over 15 traits**

**Dataset**	**G(A)**	**G(A + X)**	**G**_**c**_**(A + X)**	**G(A) + G(X)**	**G(A) + Pol**	**G**_**c**_**(A + X) + Pol**
54K_real	38.0	38.5	38.5	38.5	38.9	39.3
IMP_test	37.9	38.3	38.3	38.4	38.9	39.2
IMP_0.5ref	37.8	38.3	38.3	38.3	38.8	39.1
LD_real	33.0	33.5	33.6	33.6	35.5	35.9

^a^54K_real: all animals with marker data from the 54K chip; IMP_test: for the test animals in genomic prediction, the 54K marker data were imputed from LD marker data; IMP_0.5ref: for half (randomly chosen) of the reference animals, the 54K marker data were imputed from LD marker data; LD_real: all animals had LD marker data without extension to the 54K marker data; ^b^G(A): model with a **G** matrix built using autosomal markers only; G(A + X): model with a **G** matrix built using all markers and treating X-specific markers as autosomal markers; G_c_(A + X): model with a **G** matrix built using all markers and specifying sex-linked inheritance of X-specific markers; G(A) + G(X): model with an autosome **G** matrix and an X chromosome **G** matrix; G(A) + Pol: model G(A) plus a residual polygenic effect; G_c_(A + X) + Pol: model G_c_(A + X) plus a residual polygenic effect.

A model that included a residual polygenic effect improved the reliability of predicted breeding values, with an average increase of about 0.8% points (Table 4). For all scenarios, the greatest improvement in reliability by including a residual polygenic effect in the model was observed for the traits longevity and other diseases. Reliability of GEBV using the LD genotypes was 5% points lower than when using the real 54K genotypes and applying models without a polygenic effect, and 3.4% points lower when applying models with a polygenic effect. Furthermore, genomic predictions based on the imputed datasets of IMP_test and IMP_0.5ref were almost as accurate as predictions based on the real 54K data.

Regression coefficients of DRP on genomic predictions based on the real 54K or imputed 54K genotype data ranged from 0.782 to 1.064, except for longevity, for which the regression coefficients ranged from 0.631 to 0.685 (Table 5). Averaged over the 15 traits, the regression coefficients were slightly closer to 1 with than without using the X chromosome markers for prediction. Regression coefficients were the same when using real versus imputed 54K genotype data. In addition, models that included a residual polygenic effect resulted in regression coefficients considerably closer to 1 than models without a polygenic effect, which indicates a reduction of prediction bias from including polygenic effects. Regression coefficients deviated more from 1 for genomic predictions based on LD genotype data than for predictions using the 54K genotype data, which indicates a larger prediction bias for the former. However, when using models with a residual polygenic effect, the regression coefficients based on LD genotypes were very close to those based on the 54K genotype data.

**Table 5:** **Regression coefficients of deregressed proofs on genomic predictions based on four datasets**^**a **^**with or without X chromosome markers, using different models**^**b **^**and averaged over 15 traits**

**Datasets**	**G(A)**	**G(A + X)**	**G**_**c**_**(A + X)**	**G(A) + G(X)**	**G(A) + Pol**	**G**_**c**_**(A + X) + Pol**
54K_real	0.881	0.885	0.885	0.885	0.918	0.919
IMP_test	0.881	0.885	0.885	0.885	0.918	0.919
IMP_0.5ref	0.881	0.886	0.885	0.886	0.920	0.922
LD_real	0.834	0.835	0.837	0.838	0.914	0.915

^a^54K_real: all animals with marker data from the 54K chip; IMP_test: for the test animals in genomic prediction, the 54K marker data were imputed from LD marker data; IMP_0.5ref: for half (randomly chosen) of the reference animals, the 54K marker data were imputed from LD marker data; LD_real: all animals had LD marker data without extension to the 54K marker data; ^b^G(A): model with a **G** matrix built using autosomal markers only; G(A + X): model with a **G** matrix built using all markers and treating X-specific markers as autosomal markers; G_c_(A + X): model with a **G** matrix built using all markers and specifying sex-linked inheritance of X-specific markers; G(A) + G(X): model with an autosome **G** matrix and an X chromosome **G** matrix; G(A) + Pol: model G(A) plus a residual polygenic effect; G_c_(A + X) + Pol: model G_c_(A + X) plus a residual polygenic effect.

Table 6 shows the reliability of genomic predictions when excluding one of four selected chromosomes or when deleting a random sample of markers. Compared to excluding the X chromosome, excluding chromosome 2 (similar to the X chromosome in length), chromosome 10 (similar to the X chromosome in number of annotated genes), and chromosome 26 (similar to the X chromosome in number of markers) led to larger losses in reliability. Excluding chromosome 10 led to the largest loss in reliability, while randomly deleting 827 markers (i.e. the same number of markers as on the X chromosome) led to no loss in reliability.

**Table 6:** **Reliability (R**^**2**^**, %) of genomic predictions based on the 54K SNPs (54K_real) excluding one chromosome or a random sample of 827 markers, averaged over 15 traits**

**Chromosome excluded**	**Chr length**	**Number of genes**	**Number of markers on the map**	**Number of markers after editing**	**R**^**2**^	**Difference from R**^**2**^_full_^*^
X-Chr	147.8	1128	1176	827	38.0	0.5
Chr. 2	137.1	1021	2829	2289	37.6	0.9
Chr. 10	104.3	1074	2206	1800	37.4	1.1
Chr. 26	51.7	437	1116	921	37.8	0.7
Random	-	-	-	827	38.5	0.0

^*^Difference from reliability (%) of genomic predictions obtained with a model that used a **G** matrix built with all markers and specifying sex-linked inheritance of X-specific markers.

The log likelihood ratio test statistics in Table 7 indicate that model (G(A) + G(X)) using both autosomal and X chromosome markers had a significantly better goodness of fit than model (G(A)) using only autosomal markers for 13 of the 15 traits, and that model (G_c_(A + X) + Pol) with a residual polygenic effect was significantly better than model (G_c_(A + X)) without a polygenic effect for 12 traits. As shown in Table 7, the variance accounted for by the X chromosome was significantly different from 0 for 10 traits, and the variance accounted for by the residual polygenic effect was significant for 13 traits. On average, the X chromosome accounted for 1.7% of the total additive genetic variance, and the residual polygenic effect for 17.2% of the total additive genetic variance.

**Table 7:** Log likelihood ratio statistics between models and the variance accounted for by the X chromosome and by residual polygenic effect, based on the real 54K dataset

**Traits**	**Log likelihood ratio**		**Variance (SE)**		**Variance %**^**e**^	
	**(A + X)/A**^**a**^	**(AX + P)/AX**^**b**^	**X-Chr**^**c**^	**Pol**^**d**^	**X-Chr**^**c**^	**Pol**^**d**^
Milk	13.46^*^	16.62^*^	1.05 (0.48)^*^	14.34 (3.74)^*^	0.9	12.0
Fat	27.34^*^	8.03^*^	1.41 (0.53)^*^	9.27 (3.51)^*^	1.3	8.4
Protein	27.07^*^	34.62^*^	1.80 (0.62)^*^	20.54 (3.74)^*^	1.5	17.3
Growth	0.00	16.87^*^	0.00 (0.28)	17.84 (4.67)^*^	0.0	13.5
Fertility	27.59^*^	33.85^*^	5.21 (1.66)^*^	42.81 (8.19)^*^	3.6	27.9
Birth index	3.93^*^	2.76^¤^	0.93 (0.68)	9.09 (6.14)^*^	0.8	7.7
Calving index	0.66	0.80	0.73 (0.86)	6.51 (7.21)^*^	0.7	5.9
Udder health	21.96^*^	18.6^*^	2.44 (0.84)^*^	16.58 (4.17)^*^	2.7	17.6
Other diseases	26.05^*^	47.93^*^	6.13 (2.13)^*^	70.01 (11.21)^*^	4.1	40.4
Body conformation	4.12^*^	5.08^*^	2.71 (1.42)^*^	15.82 (7.46)^*^	2.2	12.7
Feet and legs	3.62^¤^	0.00	2.16 (1.60)	0.00 (9.97)	1.5	0.0
Udder conformation	9.60^*^	0.05	2.52 (1.10)^*^	1.34 (5.76)	1.8	1.2
Milking ability	9.97^*^	10.40^*^	2.57 (1.28)^*^	23.66 (8.01)^*^	1.2	11.0
Temperament	5.23^*^	22.22^*^	3.36 (1.78)^*^	43.94 (10.30)^*^	2.5	29.8
Longevity	3.87^*^	118.57^*^	1.07 (0.97)	87.50 (9.37)^*^	0.8	53.4
**Average**	**12.10**	**22.43**	**2.27 (1.08)**	**25.28 (6.90)**	**1.7**	**17.2**

^a^Log likelihood ratio of model G(A) + G(X) to model G(A), where G(A) was the model with an autosomal **G** matrix and G(A) + G(X) was the model including an autosome **G** matrix and an X chromosome **G** matrix; ^b^Log likelihood ratio of model G_c_(A + X) + Pol to model G_c_(A + X), where G_c_(A + X) was the model with a **G** matrix built using all markers and G_c_(A + X) + Pol included also residual polygenic effect; ^c^Variance accounted by the X chromosome and estimated from model G(A) + G(X); ^d^Variance of residual polygenic effect and estimated from model G_c_(A + X) + Pol; ^e^Variance in proportion to total additive genetic variance; ^*^Significant at P < 0.05, where P was calculated as P($\chi_{\mathrm{df}=1}^{2}$); ^¤^Significant at P_m_ < 0.05, where P_m_ was calculated as 0.5P($\chi_{\mathrm{df}=1}^{2}$), e.g., when P < 0.05, P_m_ < 0.025.

Table 8 presents reliabilities of genomic predictions for each trait using models G(A), G(A) + G(X) and G_c_(A + X) + Pol, based on the 54K_real dataset and shows that the contribution of X chromosome markers to the reliability of genomic predictions differed between traits. An increase in reliability of around 2% points was observed for fertility and other diseases. Correspondingly, the variances explained by the X chromosome were much higher for these two traits than for the other traits. Longevity also showed a significant benefit of including X chromosome markers, although the variance accounted for by the X chromosome was small for this trait. Averaged over the 15 traits, including the X chromosome improved the prediction reliability by 0.5% points.

**Table 8:** Correlation between genomic predictions and deregressed proofs and reliability of genomic predictions for each trait, based on the real 54K dataset

**Traits**	**Correlation**			**Reliability %**		
	**G(A)**	**G(A) + G(X)**	**G**_**c**_**(A + X) + Pol**	**G(A)**	**G(A) + G(X)**	**G**_**c**_**(A + X) + Pol**
Milk	0.674^a^	0.676^a^	0.681^b^	48.7	48.9	49.6
Fat	0.663^a^	0.667^ab^	0.670^b^	47.1	47.6	48.0
Protein	0.655^a^	0.657^a^	0.666^b^	45.9	46.2	47.5
Growth	0.665^a^	0.665^a^	0.668^a^	47.2	47.2	47.6
Fertility	0.520^a^	0.532^b^	0.538^b^	40.7	42.6	43.5
Birth index	0.517^a^	0.518^a^	0.518^a^	32.5	32.7	32.7
Calving index	0.452^a^	0.454^a^	0.452^a^	30.3	30.5	30.2
Udder health	0.563^a^	0.568^a^	0.569^a^	39.5	40.1	40.3
Other diseases	0.447^a^	0.459^b^	0.481^c^	36.3	38.2	41.9
Body conformation	0.480^a^	0.478^a^	0.480^a^	27.6	27.4	27.6
Feet and legs	0.452^a^	0.456^a^	0.457^a^	33.2	33.7	33.9
Udder conformation	0.595^a^	0.598^a^	0.598^a^	44.0	44.5	44.4
Milking ability	0.642^a^	0.644^a^	0.644^a^	47.1	47.4	47.3
Temperament	0.342^a^	0.342^a^	0.348^a^	18.3	18.3	19.0
Longevity	0.463^a^	0.468^b^	0.494^c^	31.1	31.8	35.4
**Average**	**0.542**	**0.545**	**0.551**	**38.0**	**38.5**	**39.3**

G(A): model with a **G** matrix built with autosomal markers only; G(A) + G(X): model with an autosome **G** matrix and an X chromosome **G** matrix; G_c_(A + X) + Pol: model with a **G** matrix built with all markers plus a residual polygenic effect; ^a,b,c^Correlations within a trait without common superscript differed significantly (P < 0.05), according to Hotelling-Williams’ t-test.

The benefit of including polygenic effects into the model also differed among traits (Table 8). A significant increase in the reliability of genomic predictions from including a residual polygenic effect was obtained for four traits. The largest improvements were for longevity (3.6%) and other diseases (3.7%). For these two traits, the variance accounted for by residual polygenic effect was more than 40% of the total additive genetic variance (Table 7). For the other traits, the average improvement in prediction reliability was 0.3%.

## Discussion

This study investigated the accuracy of genotype imputation for markers on the X chromosome and the impact of including X chromosome markers on reliability of genomic predictions. The results showed that averaged over the 15 traits evaluated, including X chromosome markers improved the reliability of genomic prediction slightly, ranging from 0.3 to 0.5% points in various datasets and using different models. The variance accounted for by the X chromosome was about 1.7% of the total additive genetic variance. Gains in reliability from including the X chromosome were smaller than observed in a previous study on USA Holstein cattle by VanRaden et al. [7], who reported an increase in reliability of 1.5%, averaged over nine traits, although the X chromosome accounted for only 1% of the total genetic variance in their study. When the genomic model included a residual polygenic effect, breeding values predicted using marker data that included X chromosome markers were still more accurate than those predicted without X chromosome markers. This means that a model that includes a residual polygenic effect does not recover the loss of prediction accuracy from exclusion of X chromosome markers.

The loss of prediction accuracy from exclusion of the X chromosome was smaller than when an autosome of similar size (chromosome 2), or with an equivalent number of annotated genes (chromosome 10), or with an equivalent number of markers (chromosome 26) was excluded. There are two possible reasons why markers on the X chromosome contribute less to the reliability of genomic predictions than these three autosomes. One reason is that the density of markers on the X chromosome is much lower than that on autosomes; the average distance between adjacent markers is about 180 kb on the X chromosome and 60 kb on the autosomes in the 54K marker data. The second reason is that markers on the X chromosome represent weaker relationships between individuals in the present data, which consisted only of males. The impact of genetic relationships between animals in the reference and test datasets on reliability of genomic predictions for test animals has been reported in many previous studies [11,20-22]. Since the relationship between sires and sons is 0 for the X chromosome, information of a sire does not directly influence the son’s GEBV explained by the X chromosome. On the contrary, information of a sire directly influences the son’s GEBV explained by the autosomes, as reported in previous studies that showed that reliability of GEBV is about 5 to 10% higher for the test animals with than without their sires in the reference population [23,24].

When a random set of 827 markers (i.e. the number of markers on the X chromosome) was excluded from the analysis, there was no loss in reliability of genomic prediction. This is explained by the fact that the effects of the removed markers are in part accounted for by other markers that are in linkage disequilibrium with the removed markers. Therefore, the loss in prediction reliability from removing a set of randomly chosen markers should be much smaller than the loss caused by removing an entire chromosome. In other words, if removing an entire chromosome leads to a larger loss in prediction reliability than removing a set of randomly chosen markers, this chromosome contributes to the reliability of genomic prediction due to linkage disequilibrium between the markers and causative genes on this chromosome. Thus, the fact that we observed a loss in prediction reliability when removing the X chromosome markers but not when removing 827 randomly chosen markers confirms that markers on the X chromosome are in linkage disequilibrium with causative genes on that chromosome which affect the traits studied.

A **G** matrix that takes the sex-linked inheritance for X-specific markers into account is expected to improve genomic prediction when using X chromosome markers, compared to a **G** matrix that deals with X-specific markers as autosomal markers. However, models G(A + X) and G_c_(A + X) gave the same reliability of genomic predictions, though the **G** matrix in model G_c_(A + X) took the sex-linked inheritance for X-specific markers into account while the **G** matrix in model G(A + X) did not. One reason for this result could be that the number of X-specific markers was too small to obtain a clear improvement in genomic predictions by correctly taking the sex-linked inheritance into account when calculating the **G** matrix. Another reason is that all animals in the current data were males, for which ignoring sex-linked inheritance in the calculation of the **G** matrix could have a small impact on relationship coefficients. Currently, in many countries and cattle populations, a large number of females are genotyped to increase the size of the reference population or to obtain their GEBV [25,26]. When genomic data that include information from males and females and the markers on the X chromosome are used, a **G** matrix that appropriately accounts for sex-linked relationships is expected to be important for genomic prediction using the GBLUP model.

Reliabilities of genomic predictions based on the imputed datasets of IMP_test and IMP_0.5ref were similar to those of predictions based on the real 54K data. This result is inconsistent with previous studies on genomic predictions using imputed 54K genotype data from a 3K marker panel in Nordic and French [27] and German Holstein populations [28], in which, on average, each 1% of imputation allele error rate resulted in a loss in prediction reliability of 1.3% points. The lower loss in reliability in our study could be due to the fact that the density of the LD chip (7K) used here was twice that of the 3K chip. Even when using the 7K genotype data without imputation, the reliability of genomic predictions was only 5.0% points lower than the reliability of predictions using the real 54K genotype data. Thus, an allele error rate of 1.2% in imputation from the 7K to the 54K marker data may have very little influence on the reliability of genomic predictions. Similarly, a previous study (Peipei Ma et al., personal communication) investigated the impact of imputation from the 54K to the 777K SNP panel by using a combined 777K reference population and reported that an improvement of the imputation error rate by about 2% did not result in a corresponding improvement in the reliability of genomic predictions. These results suggest that the impact of imputation accuracy on genomic prediction not only depends on imputation accuracy, but also on the number of markers in the lower density panel.

A model that included a residual polygenic effect increased the reliability of genomic predictions by 0.8% points on average across the 15 traits. This was larger than the 0.3% point increase reported by Gao et al. [29] for the same population. However, the present study estimated residual polygenic variance for each trait, while in Gao et al. a constant ratio of residual polygenic variance to total additive genetic variance was used for all traits. The estimated ratios of residual polygenic variance to total additive genetic variance ranged from 0 to 53.4% among the 15 traits studied here. These results indicate that trait-specific weights on residual polygenic effects should be used in genomic prediction, instead of a constant weight across traits. Furthermore, a model that included a residual polygenic effect reduced prediction bias, which was in line with the results reported by Liu et al. [30] and Gao et al. [29]. In practical genetic evaluations, GEBV are usually blended with the EBV from the conventional pedigree-based BLUP model. It is necessary to investigate whether the predicted genomic breeding values that include a residual polygenic effect result in double counting when blending them with traditional EBV. This could occur because the residual polygenic effect is already included in the GEBV, and the blending procedure uses the residual polygenic effect once again.

Accuracy of imputation from the 7K to the 54K marker panel was high (allele error rate of 1.2% using Beagle), which was in line with previous studies [5,31]. Imputation accuracy was lower for markers on the X chromosome than for markers on autosomes, which is probably mainly due to the fact that the density of markers was lower on the X chromosome than on autosomes. The average interval between adjacent markers on the X chromosome was three times as large as that on autosomes in the 54K data, and was nearly twice as large in the 7K data. Moreover, markers in the PAR had much lower imputation accuracy than X-specific markers, although the markers on the PAR were about twice as dense as X-specific markers in both the 7K and the 54K data. This can be explained by the fact that the PAR is a small segment (about 11 Mbp based on our estimation), which could reduce imputation efficiency. Another explanation could be that X-specific markers may have lower recombination rates than PAR markers, since crossovers occur only in females. Poor imputation accuracy for PAR markers was also reported by Johnston et al. [6] in the imputation from the 3K to the 54K panel.

## Conclusions

Although the accuracy of genotype imputation for markers on the X chromosome was lower than that for autosomal markers, the accuracy of imputation from the 7K to the 54K panel for markers on the X chromosome was still high in the Nordic Holstein population. Including markers on the X chromosome slightly increased the reliability of genomic predictions. Based on our data which included only bulls, using a **G** matrix that took the sex-linked inheritance of X-specific markers into account did not improve prediction compared to a **G** matrix that did not. Although the improvement in the reliability of genomic prediction obtained from the X chromosome is small, including X chromosome markers does not result in any extra cost. Therefore, it is recommended to use markers on the X chromosome for genomic evaluation.

## Competing interests

The authors declare that they have no competing interests.

## Authors’ contributions

GS, BG and MSL conceived and designed the study. GS performed the analysis and wrote the manuscript. BG, IS, GAP and MSL helped in interpreting results and improving the manuscript. All authors read and approved the manuscript.
